# Facile Synthesis of Magnetic Nitrogen-Doped Porous Carbon from Bimetallic Metal–Organic Frameworks for Efficient Norfloxacin Removal

**DOI:** 10.3390/nano8090664

**Published:** 2018-08-26

**Authors:** Hui Wang, Xi Zhang, Yan Wang, Guixiang Quan, Xiangyun Han, Jinlong Yan

**Affiliations:** 1 School of Environmental Science and Engineering, Yancheng Institute of Technology, Yancheng 224051, China; whsl@ycit.cn (H.W.); shanliangbang@163.com (Y.W.); qgx@ycit.cn (G.Q.); hxy16_2000@163.com (X.H.); 2 College of Life and Environmental Science, Shanghai Normal University, Shanghai 200234, China; m18321255826_1@163.com

**Keywords:** self-catalytic pyrolysis, porous carbon, metal–organic frameworks, antibiotics, adsorption

## Abstract

Magnetic nitrogen-doped porous carbon (MNPC) has been prepared via self-catalytic pyrolysis of bimetallic metal-organic frameworks (MOFs). The as-obtained MNPC showed favorable features for antibiotics adsorption such as high specific surface area (871 m^2^ g^−1^), high pore volume (0.75 cm^3^ g^−1^), porous structure, good graphitization degree, and rich N-doping. Moreover, the MNPC has magnetic properties due to the Co species, which is embedded with a high dispersion, so the absorbent can be easily separated. Based on the above excellent characteristics, the MNPC was used as the absorbent for norfloxacin (NOR) removal. The experimental maximum NOR adsorption capacity of MNPC was 55.12 mg g^−1^ at 298.15 K and a pH of 6.0 with an initial NOR concentration of 50 mg L^−1^. The data analysis of the kinetics revealed that the experimental data of NOR uptakes versus time agreed with the pseudo-second order model. The isotherm data analysis revealed the favorable application of the Freundlich model. Based on the adsorption results over a wide range of conditions, the dominant adsorption mechanisms were found to be pore-filling, electrostatic interaction, and the H-bond.

## 1. Introduction

Over the past few decades, the emission of pharmaceutical compounds into the environment has sharply increased due to fast population growth and the rapid expansion of the pharmaceuticals industry. Antibiotics are one of most important type of pharmaceuticals, and are usually used as drugs or feed additives [1,2,3,4]. However, large amounts of antibiotics are stable and cannot be easily degraded, thus they are persistent in the environment. In addition, antibiotics could generate antibiotic-resistance genes in microorganisms, which can proliferate and widely disseminate in ecosystems. Fluoroquinolones are a commonly used antibiotic and their concentration is relatively high in the environment [5,6]. Norfloxacin (NOR) is one of the most frequently used fluoroquinolone antibiotics, and is always used to treat infectious diseases. It has been detected in the surface water and found to be toxic to aquatic organisms and human beings [7,8]. Therefore, it is necessary to develop a cost-effective method to remove NOR from wastewater.

Such methods as advanced oxidation, electrochemical methods, and biological treatments have been applied extensively to remove NOR from wastewaters. Among all of these methods, adsorption is the top priority owing to its simplicity, low operating cost, safety, and efficiency [9,10,11]. With such advantages as a large specific surface area and porous structure, carbon materials have been applied to remove NOR from water. For example, Xing et al. investigated the adsorption of norfloxacin (NOR) onto multiwall carbon nanotubes and activated carbon, and the results showed that activated carbon (AC) has a better NOR sorption capacity due to its higher surface area [12]. Theydan et al. prepared AC from a lignocellulosic biomass to remove NOR from water, and a maximum removal percentage of 98.13% was achieved [13]. Although a significant amount of research has been expended on adsorbing materials for antibiotics removal during the past few decades, development of novel adsorbents with higher performance is still needed.

As a new class of porous inorganic–organic materials, metal–organic frameworks (MOFs), have attracted wide attention owing to their high surface area and tunable pore size, which is widely used in areas of drug delivery, gas storage, and separation and catalysis [14,15,16,17]. Recently, MOFs have been used as templates or novel sources to prepare porous carbons through further carbonization. For example, Xu et al*.* applied MOFs as sacrificial templates to synthesis nanoporous carbons for the first time. They introduced furfuryl alcohol into the MOF-5 through a vapor phase protocol, which was then carbonized at 1000 °C under an inert atmosphere to obtain porous carbon [18]. Park et al. presented hierarchically porous carbon from highly crystalline MOFs and used it as a hydrogen storage adsorbent [19]. Most recently, Huang et al. demonstrated the application of MOF-derived porous carbon as an adsorbent for antibiotics removal. They prepared porous carbon through a one-step carbonization of zeolitic imidazolate framework-8 (ZIF-8), which showed a larger specific surface area due to the vaporization of the center metal (Zn) of ZIF-8 during the pyrolysis process. They were further used for ciprofloxacin removal from water [20]. Although porous carbon derived from ZIF-8 has a high specific surface area for antibiotics adsorption, it has limitations in terms of adsorption capacity and ease of separation. 

Recently, the sustainability of the adsorption process has been advocated, such as: green adsorbent and green separation methods [21,22,23]. It has been demonstrated that the incorporation of magnetic nanoparticles on the surface of adsorbents can be engineered to allow the magnetic separation and recovery of the absorbents [24,25]. Wang et al. synthesized reduced graphene oxide/magnetite composites through an in situ reaction and utilized it as an adsorbent with a magnetically separable property for fluoroquinolone antibiotics [26]. Cai et al. encapsulated magnetic nanoparticles into carbon with a well-constructed core-shell structure, and then used it as an adsorbent for organic pollutants isolation [27]. However, the preparation of magnetic adsorbents usually needs additional processes to load the magnetic metal oxide, and it is difficult to control the dispersion of loaded particles during the synthesis process. 

**Figure 1:**
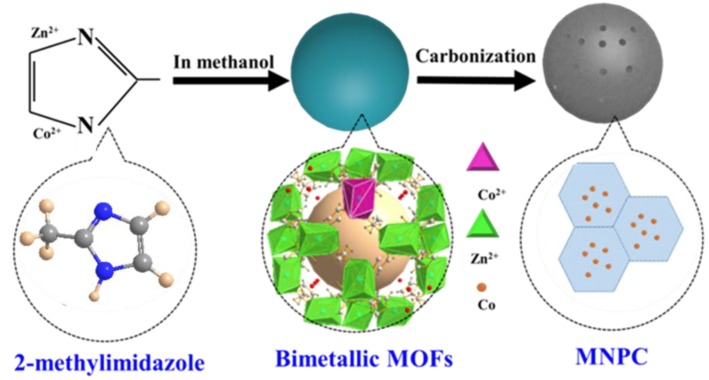
Schematic illustration of the construction process for the magnetic nitrogen-doped porous carbon (MNPC).

Herein, we report a simple but efficient solution process for the fabrication of a new form of magnetic nitrogen-doped porous carbon (MNPC) adsorbents for the NOR removal. The MNPC was directly synthesized by self-catalytic pyrolysis of bimetallic MOFs, which were prepared by using divalent Zn^2+^ and Co^2+^ as center metal ions and 2-methylimidazole as the ligand (Figure 1). In the carbonization process, the Zn, with a boiling point of around 900 °C, was evaporated during the calcination process, and the porous structure was formed simultaneously. Furthermore, the Co species were embedded in the porous structure with a high dispersion due to the coordination structure of the MOF’s precursor, and so the MNPC had magnetic properties. Moreover, the Co species can act as catalyst to improve the graphitization degree of MNPC, which can enhance the adhesion between antibiotics and adsorbents through π–π conjugation. More importantly, by the development of such multiple structures, the adsorption performance was significantly enhanced.

## 2. Materials and Methods 

### 2.1. Synthesis of MNPC

Materials: The zinc nitrate hexahydrate (Zn(NO_3_)_2_·6H_2_O), cobaltous nitrate hexahydrate (Co(NO_3_)_2_·6H_2_O), 2-methylimidazole and norfloxacin were analytic grade provided by Aladdin Chemical Reagent Co., Ltd., Shanghai, China. 

Synthesis of MNPC: Typically, Co(NO_3_)_2_·6H_2_O (0.27 g, 0.9 mmol) and (Zn(NO_3_)_2_·6H_2_O) (1.40 g, 4.7 mmol) were first dissolved in 100 mL of methanol. 2-Methylimidazole (3.70 g, 45.1 mmol) in 100 mL methanol was then added to the above solution. After quickly stirring for 24 h, the products were separated by centrifugation and washed thoroughly with methanol. The obtained bimetallic MOFs were dried at 50 °C overnight, and further activated at 200 °C for 24 h under a vacuum before use. The as-synthesized bimetallic MOFs nanocrystals were heated to 950 °C with the ramp rate of 3 °C/min under a N_2_ atmosphere and carbonized at 950 °C for 2 h, and then cooled to room temperature naturally. Finally, the MNPC was entirely fabricated. The magnetic carbon (MC) prepared by MOFs with only Co ions as a central ion was used for comparison. The porous carbon (PC) prepared by MOFs with only Zn ions as a central ion was also prepared. In fact, the methanol used in this process could be recycled through membranes to realize sustainable fabrication [28,29]. 

### 2.2. Adsorption Performance of MNPC

The adsorption experiments on NOR were conducted in 250 mL stopper conical flasks, and then placed in a thermostatic shaker with a speed of 200 rpm. In the adsorption experiment, 80 mg of adsorbent was added to 100 mL of adsorbate solution. The influence of initial concentrations (5–50 mg L^−1^), pH (2–10), temperature and ionic strength on the adsorption of NOR were also investigated. The solution pH was adjusted by dilute HCl or NaOH solution. The concentration of NOR was measured with a UV–Visible spectrophotometer (TU-1810, Beijing Purkinje General Instrument Co. Ltd., Beijing, China) at 272 nm. The adsorbed capacity (*q*) and removal rate (*η*) were calculated according to the following equations:

*q* = (*C*_0_ − *C*_t_)*V*/*m* (1)

*η* = (*C*_0_ − *C*_t_)/*C*_0_ (2)

where *C*_0_ and *C*_t_ (mg L^−1^) represent the initial and final concentrations of NOR in the feed solution, respectively, *V* is the volume of NOR solution (L), and *m* is the dry mass of MNPC (g).

## 3. Results

### 3.1. Characterization

**Figure 2:**
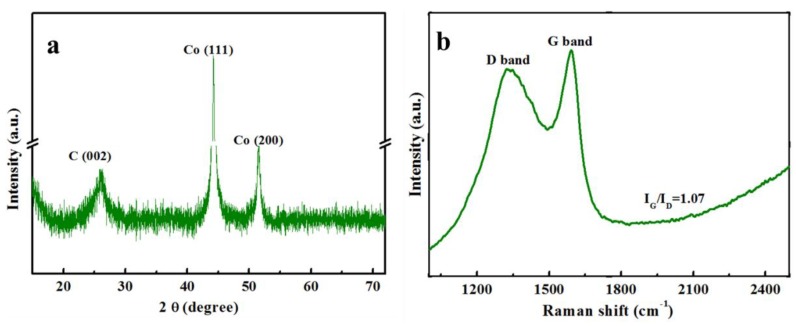
(**a**) X-ray diffraction (XRD) pattern and (**b**) Raman spectrum of the MNPC.

The X-ray diffraction (XRD, Rigaku D/Max-RB, Rigaku Corporation, Tokyo, Japan) measurements is usually conducted to evaluate the structure of materials, and the XRD pattern of the MNPC is presented in Figure 2a. The MNPC shows an obvious diffraction peak at the 2θ = 26°, corresponding to the (002) plane of the graphitic carbon [30]. The diffraction peaks located at around 44° and 51° are ascribed to *fcc* Co, which is embedded in the carbon shell [31,32]. There was no characteristic peak of Zn in the XRD patterns due to the effective evaporation during the high-temperature calcination. The XRD pattern of MC was similar to that of MNPC (Figure S1). The graphitization degree of MNPC was further detected using Raman spectra (JY H800UV, Jobin-Yvon Corporation, Longjumeau, France), and the result is shown in Figure 2b. Two broad peaks at 1330 and 1583 cm^−1^ are obvious, and are related to the D-band and G-band, respectively. The D-band is associated with defects in the carbon structure, while the G-band is attributed to the vibration of sp^2^ carbon atoms in both the rings and chain [25]. The graphitization degree of MNPC can be found by calculating the ratios of the integrated intensities of the graphitic G-band to that of the disorder-induced D-band. As calculated, the value of *I*_G_/*I*_D_ was 1.07, which was close to the MC (1.02, Figure S2a). However, the values of *I*_G_/*I*_D_ were higher than that of PC (0.94, Figure S2b) due to the catalytic action of Co.

**Figure 3:**
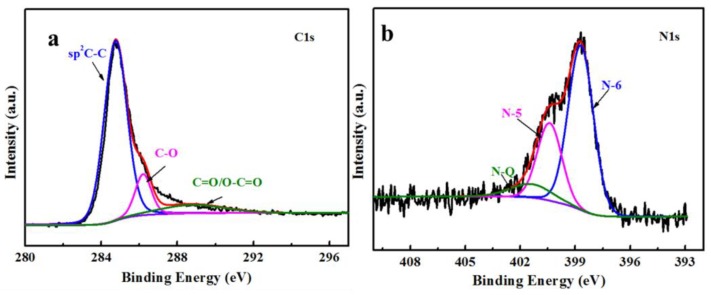
(**a**) C 1s spectra and (**b**) N 1s spectra of the MNPC.

To further probe for the chemical identification of elements in the MNPC, the X-ray photoelectron spectroscopy (XPS, PHI-5000C ESCA system, Perkin–Elmer, Hopkinton, MA, USA) measurements were performed. According to the results, the elemental content of C, N, O, and Co was 80.29, 10.58, 7.19, 1.95 at %, respectively. The C, N, O, and Co contents of MC are 90.67, 3.6, 4.38, and 1.34 at %, respectively (Figure S3). The high-resolution C1s spectra (Figure 3a) could be fitted with three peaks at 284.6, 286.4, and 287.8 eV, corresponding to the sp^2^ aromatic rings, C–O, and C=O, respectively [33]. The peak of sp^2^ carbon showed the strongest intensity, indicating that the MNPC predominantly consisted of sp^2^-hybridized carbon due to the effective catalytic graphitization. As seen from the high-resolution N1s (Figure 3b), three different types of nitrogen species were well deconvoluted. The N-6 atoms were located at 398.5 eV, and were bonded with two carbon atoms in a C_6_ ring, so a pair of lone electrons could be introduced simultaneously. This was beneficial to the formation of a hydrogen bond with the NOR molecule. The N-5 was centered at 400.4 eV, associated with the adjacent phenolic or carbonyl group. The N–Q atoms bond with three carbon atoms in the center of graphitic plane [34,35]. The additional N-doping can increase the adsorption sites for NOR, and further improve the adsorption performance of MNPC.

**Figure 4:**
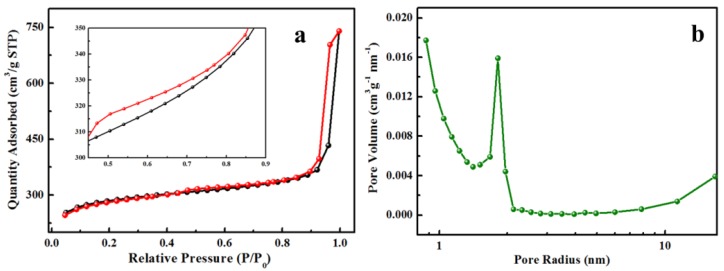
(**a**) N_2_ sorption isotherm and (**b**) pore size distribution of the MNPC.

The N_2_ sorption isothermal (ASAP 2020, Micromeritics Inc., Norcross, GA, USA) was further examined to analyze the pore structure of MNPC. As seen from Figure 4a, the MNPC showed a typical IV-type isotherm with a hysteresis loop at *p*/*p*_0_ = 0.4–1.0 (inset), indicating the mesoporous structure of MNPC [36]. Figure 4b shows the Barrett–Joyner–Halenda (BJH) pore size distribution profile of MNPC derived from desorption branches of isotherms. Clearly, the MNPC pores’ radii was mainly concentrated at 2.0 nm, further indicating that the mesopores were dominant in the structure of MNPC. The specific surface area of MNPC was 871 m^2^ g^−1^, much larger than that of MC obtained with the absence of a Zn ion (Figure S4). With Zn coordination, the ZnO would be formed during the carbonization process, which can act as sacrificial template accelerating the formation of the porous structure of MNPC [37,38]. Furthermore, the pore volume of MNPC was 0.76 cm^3^ g^−1^ (Figure 4b), which is much larger than that of MC (0.16 cm^3^ g^−1^). The increased specific surface area and pore volume are favorable for increasing the accessible surface area for NOR accumulation during the adsorption process and then enhance the adsorption capacity.

**Figure 5:**
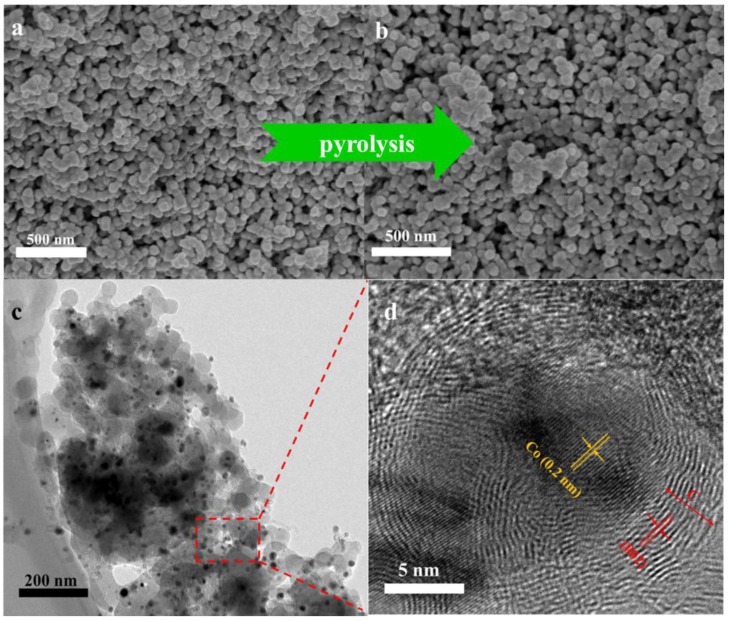
SEM images of (**a**) Bimetallic metal-organic frameworks (MOFs) precursor; and (**b**) the MNPC; (**c**) TEM and (**d**) HRTEM images of the MNPC.

The surface morphology of MNPC was investigated with scanning electron microscopy (SEM, JEOL JSM-6700F, Tokyo, Japan) and transmission electron microscopy (TEM, JEOL JEM-200CX, Tokyo, Japan). As seen from the SEM image in Figure 5a, the bimetallic MOFs precursor shows a cubic-like structure with an average size of 50 nm. After pyrolysis at 950 °C, the Zn species were volatilized, and the pores left simultaneously [38]. Moreover, the MNPC retained the morphology of the MOF’s precursor with a good dispersion (Figure 5b). This indicates that the structure kept well, even after the high-temperature calcination. The TEM image in Figure 5c reveals that the MNPC had a uniform morphology with an interconnected porous structure, and the Co nanopaticals (NPs) were embedded in the porous carbon. The high-resolution transmission electron microscopy (HRTEM) image (Figure 5d) shows further that MNPC exhibits an obvious core-shell structure. The graphitic carbon structures were the shells with an interplane spacing of (002) crystal lattice (3.4 Å), which resulted from the catalytic graphitization behavior of Co NPs [31]. Moreover, the Co NPs were tightly wrapped by graphitic carbon shells due to the coordinating structures of bimetallic MOFs as a precursor. In addition, the HRTEM image shows a distinct lattice fringe with an interplanar spacing of 0.2 nm, which matched well with the spacing of (111) planes of the Co phase. Besides, the MC showed a dodecahedron-like structure with a particle size around 250 nm, and the Co also embedded in the carbon structure (Figure S5). 

**Figure 6:**
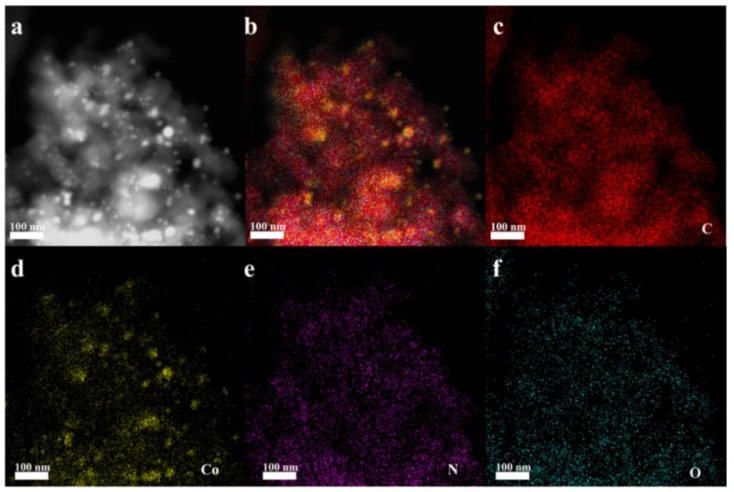
The high-angle annular dark field-scanning electron microscopy (HAADF-STEM): (**a**) image, and (**b**–**f**) mapping images of MNPC.

As seen from the high-angle annular dark field-scanning electron microscopy (HAADF-STEM) (Figure 6a), The Co NPs were embedded into graphitic carbon structure. The elemental mapping was performed to illustrate the spatial distribution of C, N, O, and Co in the structure of MNPC in Figure 6b. As revealed in Figure 6c–f, the elemental mapping results further confirmed the uniform distribution of Co and N species within the MNPC structure. Besides, the MC also showed a homogeneous distribution (Figure S5). It is generally accepted that the N species can promote the formation of hydrogen bonds and accelerate the adsorption performance. Moreover, the Co species with a good dispersion within the carbon structure is beneficial to the further separation of adsorbents. 

### 3.2. Adsorption Performance

**Figure 7:**
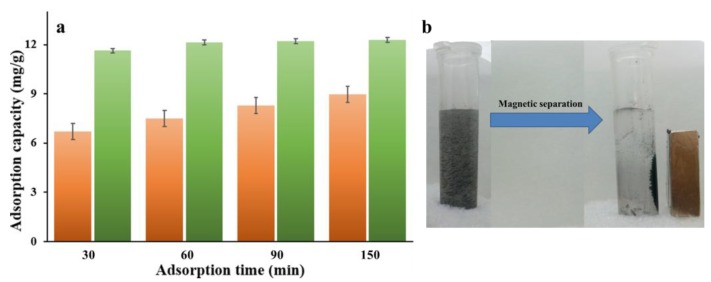
(**a**) Plots of adsorption capacity vs adsorption time of MNPC and MC in the NOR aqueous solutions at concentrations of 10 mg L^−1^; and (**b**) the photo of MNPC separated under an external magnetic field.

The adsorption behavior of the MNPC on the NOR was investigated by batch mode experiments in 10 mg L^−1^ aqueous solution. As seen from the adsorption curves in Figure 7, the adsorption capacity sharply increased with the adsorption time, suggesting that the NOR in the aqueous solution could be quickly and easily removed by the adsorbents [39]. As time goes on, the change of adsorption capacity became slower until reaching an adsorption equilibrium owing to the fact that the number of adsorption sites decreased as the adsorption time increased. Obviously, the adsorption capacity of the MNPC adsorbents was much larger than that of MC, indicating that the MNPC exhibited much better adsorption performance. After 150 min, the final adsorption capacity of MNPC adsorbents was 8.84 mg g^−1^, larger than that of MC (7.98 mg g^−1^). As seen from the Figure 7b, the MNPC in the aqueous solution could be easily separated under an external magnetic field. 

The better adsorption performance of MNPC adsorbents can be ascribed to the following aspects: First, the larger specific surface area of MNPC can provide more adsorption sites for the NOR adsorption. Second, the NOR molecules can be easily transported between the smooth channels in the MNPC due to the interconnected porous structure. Moreover, the MNPC has a good graphitization degree, which is beneficial to the formation of π–π interactions between NOR and absorbents, which then further improves the adsorption capacity [8,40]. Furthermore, hydrogen bonding is easy to form between MNPC adsorbents and NOR due to the effect of nitrogen doping, which further promotes adsorption performance [38]. In conclusion, combining the pore structure, large specific surface area, good graphitization degree, and the effective nitrogen doping, MNPC can be considered as an excellent candidate material for NOR adsorption application.

As is well known, the structure and surface properties of adsorbents have important influence on the adsorption performance. Generally, the adsorbent’s structure has a great effect on the physical adsorption, and the chemical adsorption is usually related to the functional groups on the surface of adsorbents [41]. The MNPC has large specific surface area, which can provide abundant adsorption sites for NOR adsorption. The porous structure is beneficial to the NOR molecules’ penetration. In addition, such oxygen-containing functional groups as –COOH and –OH and N-doping are on the surface of MNPC, so the hydrogen bonding can be easily formed between the NOR molecules the MNPC, which then promotes the adsorption capacity [8]. Moreover, the aromatic structures and C=C double bonds in NOR can contribute to the affinity between MNPC and NOR through the π–π interactions and then increase the adsorption capacity [42]. 

**Figure 8:**
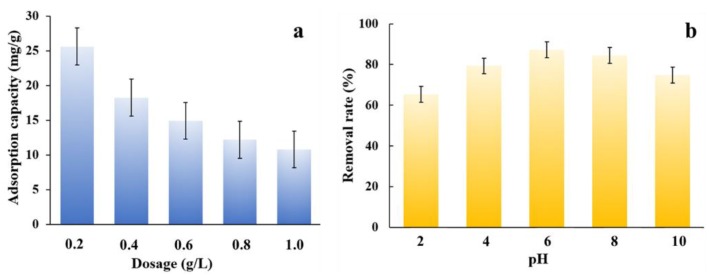
(**a**) Plots of adsorption capacity vs dosage; and (**b**) plots of removal rates vs. pH value with a dosage of 0.8 g L^−1^. All the curves were obtained in a 10 mg L^−1^ NOR aqueous solution at 30 °C.

As is well known, the amount of adsorbents has a critical effect on the adsorption performance. The influence of the adsorbent’s dosage was explored by adding various amounts of MNPC to 100 mL of a 10 mg L^−1^ NOR solution. As seen from Figure 8a, the adsorption capacity decreased with the increase of the absorbent’s dosage due to the completely exposed adsorption sites at the low dosage. While at higher dosage, the unoccupied adsorption sites were excess and resulted in a lower adsorption capacity [43]. Considering the adsorbent amounts and adsorption capacity, an 0.8 g/L dosage of MNPC was selected for further studies. 

pH is another important factor affecting adsorption performance. As seen in Figure 8b, the NOR adsorption on MNPC initially increased with the pH value ranging from 2.0 to 6.0, and then decreased when the solution pH value was higher than 6.0. The NOR contained a carboxyl and piperazinyl group, which shows two proton-binding sites. Its two acid dissociation constant pKa values were 6.22 and 8.51, respectively. In the solution, the protonation–deprotonation reaction of NOR would occur. The NOR can exist in cationic form (pH < 6.2), zwitterionic/neutral form (6.2 < pH < 8.5), or anionic form (pH > 8.5) [8,12]. In acidic conditions, a large amount of H^+^ ions surrounds the surface of MNPC, which could compete with the NOR molecule existing in the cationic form, and so the binding of NOR to adsorbent is restricted. When the pH value ranges from 6.0 to 8.7, the ratio of the zwitterion form is increased, so the competition between H^+^ and NOR ions for surface adsorption sites is decreased correspondingly, resulting in an improved adsorption capacity. However, when the pH is higher than the pKa_2_ of NOR, the anionic form dominates and the repulsion between the NOR molecule and the negatively charged MNPC is increased, and so the adsorption capacity is significantly decreased. The further studies were conducted at the optimum pH value of 6.0.

**Figure 9:**
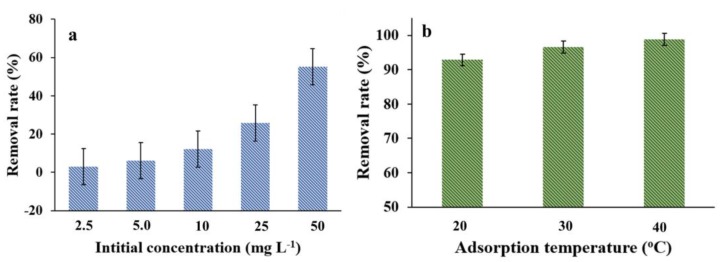
(**a**) The change of adsorption capacity vs initial concentration; and (**b**) the change of adsorption rates vs. temperature. All the curves were obtained in NOR solution with a dosage of 0.8 g L^−1^ at a pH of 7.0.

The initial NOR concentration is another key factor controlling the adsorption performance of MNPC, as shown in Figure 9a. The initial NOR concentration ranged from 1.0 to 100 mg L^−1^ at a pH of 6.0. Obviously, the adsorption capacity is increased with the solution concentration, which was increased from 3.04 to 55.12 mg g^−1^. A higher initial NOR concentration meant a higher concentration gradient, which led to a high driving force, and so the NOR molecules could quickly transfer to the pores of the MNPC. As shown in Figure 9b, the adsorption capacity of MNPC for NOR increased with increased temperature (20–40 °C), suggesting that the higher temperature was beneficial to the adsorption process.

**Figure 10:**
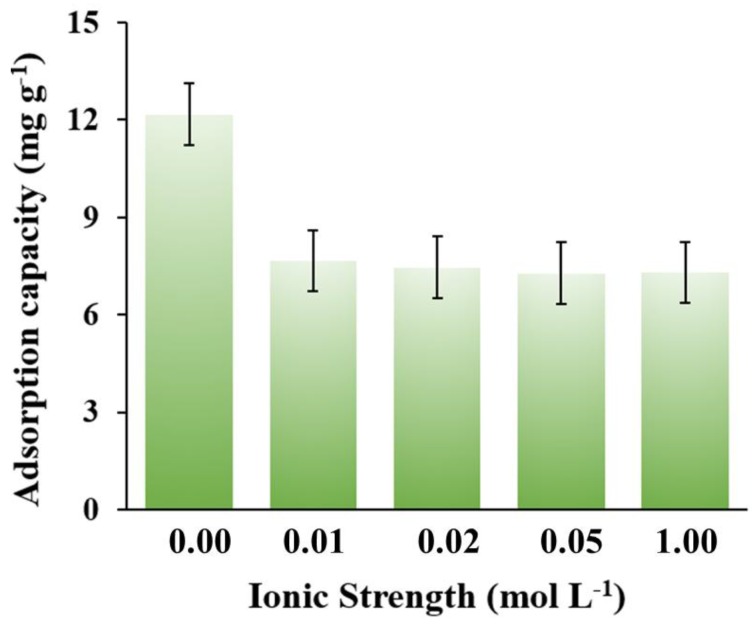
Plots of adsorption capacity vs ionic strength. All the curves were obtained in 10 mg L^−1^ NOR aqueous solution with a dosage of 0.8 g L^−1^ at a pH of 7.0.

The influence of ionic strength on the adsorption performance was also investigated and the results are shown in Figure 10. When the salt concentration increased from 0.0 to 0.1 M, the adsorption capacity decreased slightly. Generally, the salt concentration had no significant effect on the adsorption capacity of NOR on the MNPC, which indicates that the interaction between NOR and MNPC was quite stable in a certain range of salt concentration.

### 3.3. Recyclability

**Figure 11:**
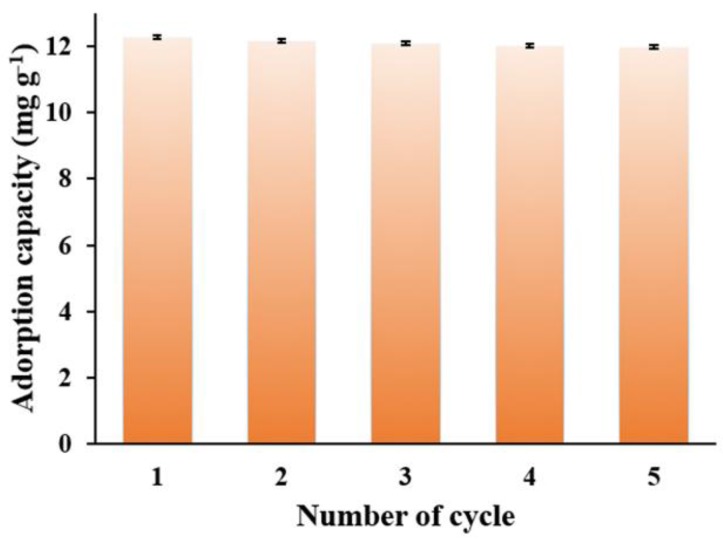
Regeneration property of the MNPC adsorbents in the 10 mg L^−1^ norfloxacin (NOR) aqueous solution with a dosage of 0.8 g L^−1^ at a pH of 7.

In practical application, the recyclability is another critical factor for the adsorbents. The recyclability of the MNPC was investigated using a methanol solution (containing 10% ammonia) as the effluents and the results are shown in Figure 11. The adsorption capacity of the MNPC remained at 12.0 mg g^−1^ after five cycles, and was slightly decreased, indicating the good regeneration performance of the absorbents in the NOR solution. Hence, the MNPC adsorbent could be reused effectively, which is helpful for reducing the cost of adsorption.

### 3.4. Adsorption Kinetics

Adsorption kinetic models are usually used to evaluate the variation of adsorption capacity with adsorption time, which can further reflect the relationship between adsorption performance and the structure of adsorbent. In this work, the pseudo-first-order and pseudo-second-order models were employed to analyze the experimental data. These two models are shown as follows:

(3) $$\;{\ln(q\;}_{e}{-q}_{t}{)\;=\;\ln q}_{e}-{\;k}_{1}t$$

(4) $$\;\frac{t}{q_{t}\;}\;=\;\frac{1}{k_{2}q_{e}^{2}\;}+\;\frac{t}{q_{e}}$$

where *q*_e_ and *q*_t_ (mg g^−1^) are NOR uptakes at equilibrium, *t* is the adsorption time, and k_1_ (min^−1^) and k_2_ (g mg^−1^min^−1^) are the rate constants of two modes [44,45]. 

**Figure 12:**
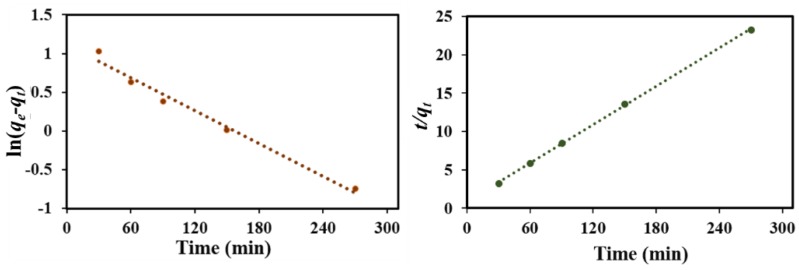
Adsorption kinetics of NOR on MNPC (**a**) pseudo-first-order and (**b**) pseudo-second-order models.

Figure 12 shows the results of fitting the two kinetic models. As seen from the Figure 11a, the experimental data severely deviates from the fitted data, and the *R*^2^ value of the pseudo-first order model was relatively low, indicating a low correlation of NOR adsorption kinetics data, and so the pseudo-first-order model was inconsistent with the experimental data. However, the experimental adsorption capacity values were in agreement with the theoretical adsorption capacity values according to the pseudo-second-order model (Figure 11b) with a corresponding *R*^2^ of 0.9996, illustrating that the adsorption data fit well to the pseudo-second-order kinetic model. 

### 3.5. Adsorption Isotherm 

**Figure 13:**
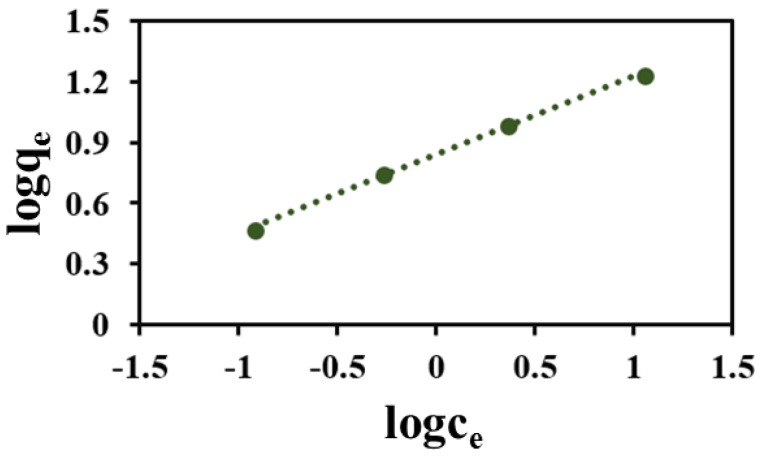
Freundlich isotherms for the adsorption of NOR by MNPC at 30 °C.

To further study how the adsorbate interacts with the adsorbent, the adsorption models have been applied to understand the adsorption mechanism. Thus the Freundlich and Langmuir adsorption isotherm models were used according to:

(5) $$\;{\;q\;}_{e}\;=\;\frac{q_{m}K_{L}C_{e}}{{1+K}_{L}C_{e}}$$

(6) $$\;q_{e}{\;=\;K\;}_{F}C_{e}^{1/n}$$

where *q*_e_, *q*_m_, and *C*_e_ (mg g^−1^) are the adsorption capacity, equilibrium concentration, and the maximum adsorption capacity, respectively, and *K*_L_, *K*_F_, and *n* are the Langmuir and Freundlich parameters [13]. Figure 13 displays both the experimental data and the fitting isotherms of the above two isotherm models. According to the results, the adsorption isotherm of NOR onto the MNPC fits Freundlich isotherm model with higher correlation coefficients *R*^2^ values (0.9988) compared with the Langmuir isotherm model (0.9841, Figure S6), indicating that the adsorption process predominantly features multilayer adsorption. 

### 3.6. Adsorption Thermodynamics

The adsorption of NOR on the MNPC was further demonstrated by evaluation of changes in the Gibbs free energy (△*G*^θ^), enthalpy (△*H*^θ^), and entropy (△*S*^θ^) as follows:

(7) $$\;\Delta G^{\theta }{\;=\;-RT\mathrm{lnK}\;}_{c}$$

(8) $$\;K_{c}\;=\;\frac{C_{A}\;}{C_{S}}$$

where R is the ideal gas constant, *T* represents absolute temperature (K), *C*_A_ and *C*_S_ (mg L^−1^) are the amount of NOR adsorbed and remained in the solution at equilibrium, respectively. After making the substituting of △*G*^θ^ = △*H*^θ^–*T*△*S*^θ^ into Equation (9):

(9) $$\;\ln\left(K_{c}\;\right)\;=\;-\frac{\Delta G^{\theta }\;}{RT}=\;-\frac{\Delta H^{\theta }}{RT}+\frac{\Delta S^{\theta }}{R}$$

The values of △*H*^θ^ and △*S*^θ^ were then calculated from the slope and intercept of the linear regression of ln*(K*_c_*)* versus 1/T [41]. As calculated, the value of △*H*^θ^ was 70.08 kJ·mol^−1^, indicating that sorption of NOR on the MNPC was an exothermic process. Moreover, the value of △*H*^θ^ was higher than 20 kJ·mol^−1^, indicating the NOR sorption onto MNPC could be mainly attributed to chemisorption. Another important thermodynamic parameter is entropy △*G*^θ^. As calculated, the △*G*^θ^ value was negative, indicating that the adsorption could occur spontaneously.

## 4. Conclusions

The MNPC was successfully prepared by self-catalytic pyrolysis of bimetallic MOF with Zn and Co as metal ions and 2-methylimidazole as a ligand. The resultant MNPC possessed a large surface area, porous structure, good graphitization, and highly dispersed N species, simultaneously. The synergistic effect of the above characteristics offered MNPC excellent adsorption performances. The MNPC exhibited a dramatic enhancement in the adsorption to NOR compared with the MC derived from the MOF with only Co as the metal ion. The adsorption capacity was 55.12 mg g^−1^ with an initial concentration of 50 mg L^−1^ at 30 °C. The pseudo-second-order and Freundlich models were a good fit for adsorption kinetics and isotherm for NOR adsorption. In the process of NOR adsorption onto the MNPC, the π–π interaction, hydrogen bonding, and pore-filling significantly improved the adsorption capability. Overall, this material is a potential adsorbent for the NOR and is expected to be used for removal of other pollutants in waste water.

## Supplementary Materials

The following are available online at http://www.mdpi.com/2079-4991/8/9/664/s1, Figure S1: XRD pattern of MC, Figure S2: Raman spectra of (**a**) MC and (**b**) PC, Figure S3: The XPS spectra of MC and MNPC, C1s and N1s of MC, Figure S4: N_2_ sorption isotherm of MC, Figure S5: (**a**) SEM, (**b**) TEM, (**c**) HRTEM and mapping images of MC, Figure S6: Langmuir isotherm of NOR adsorption on the MNPC at 30 °C.
