# Supplementary material for: Facile Synthesis of Magnetic Nitrogen-Doped Porous Carbon from Bimetallic Metal–Organic Frameworks for Efficient Norfloxacin Removal
Source: Nanomaterials (Basel). 2018 Aug 26;8(9):664. doi: 10.3390/nano8090664 (PMC6165088; doi:10.3390/nano8090664)
Supplement: Supplementary file 1 [file nanomaterials-08-00664-s001.pdf]

Facile Synthesis of Magnetic Nitrogen-Doped Porous Carbon from Bimetallic Metal–Organic Frameworks for Efficient Norfloxacin Removal

[1] Yancheng Institute of Technology, School of Environmental Science and Engineering, Yancheng, 224051 China; whsl@ycit.cn (H.W.); shanliangbang@163.com (Y.Z.); qgx@ycit.cn (G.Q.); hxy16_2000@163.com (X.H.);
[2] Collage of Life and Environmental Science, Shanghai Normal University, Shanghai, 200234 China; m18321255826_1@163.com
* Correspondence: yjlyt@ycit.cn; Tel.: +86-515-8829-8805

Characterization

X-ray diffraction (XRD) measurements were taken on a Rigaku D/MAX-RB X-ray diffractometer using Cu Kα radiation (40kV, 30 20 mA) and a secondary beam graphite monochromator. The Raman spectra were recorded on a spectrometer (JY H800UV) with the 633 nm laser. A transmission electron microscopy (TEM, JEOL, JEM-200CX) and scanning electron microscopy (SEM, JEOL, JSM-6700F) were well used to evaluate morphologies, and powdered samples were dispersed in ethanol by ultrasonication for 15 min in an ultrasonic bath. Nitrogen sorption isotherms were measured with an ASAP 2020 (Micromeritics) at 77 K after being degassed overnight at 593 K in a vacuum line. The Brunauer–Emmett–Teller (BET) method was utilized to calculate the specific surface areas, and the pore size distributions were derived from the desorption branches of the isotherms using the Barrett–Joyner–Halenda (BJH) model. The X-ray photoelectron spectroscopy (XPS) was recorded on a Perkin-Elmer PHI 5000C ESCA system equipped with a dual X-ray source, using the 45 MgKα (1253.6 eV) anode and a hemispherical energy analyzer.

Results and Discussion

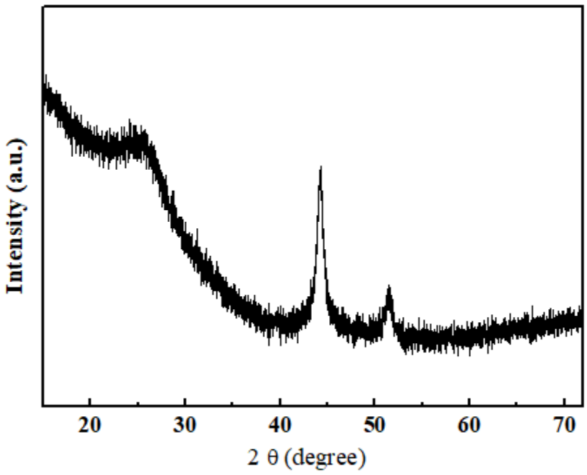

Figure S1. XRD pattern of MC.

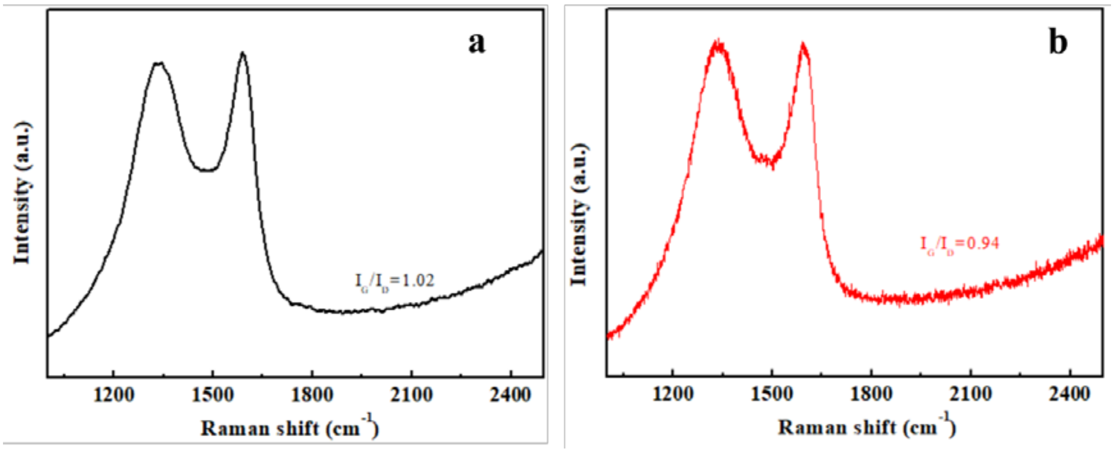

Figure S2. Raman spectra of (**a**) MC and (**b)** PC.

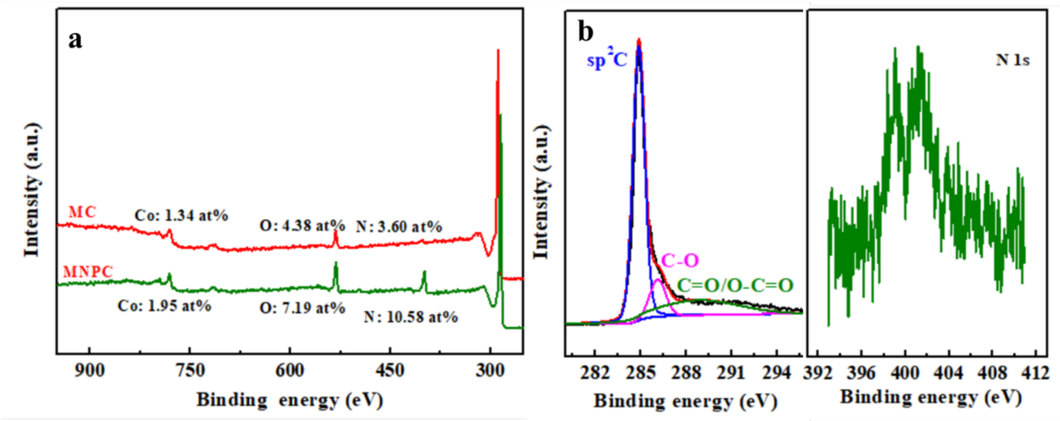

Figure S3. (**a**) The XPS spectra of MC and MNPC, (**b**) C1s and N1s of MC.

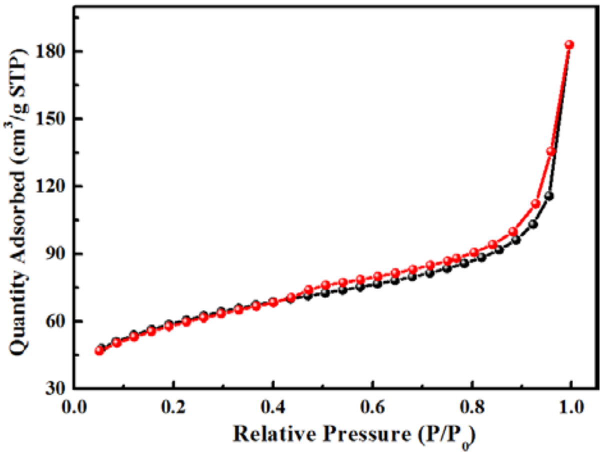

Figure S4. N_2 sorption isotherm of MC.

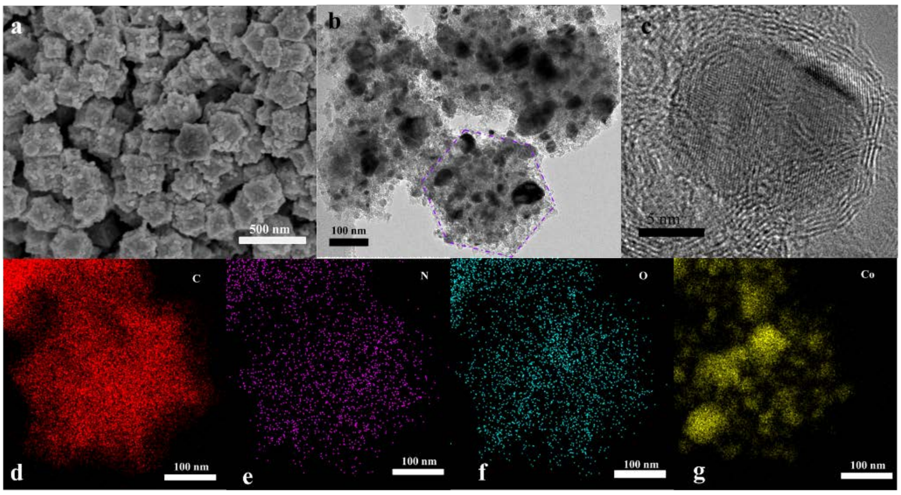

Figure S5. (**a**) SEM, (**b**) TEM , and (**c**) HRTEM and mapping images of MC.

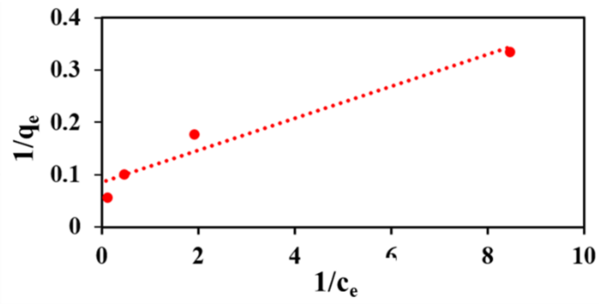

Figure S6. Langmuir isotherm of NOR adsorption on the MNPC at 30 °C.
